# Mosaic Somatic Gene Recombination as a Potentially Unifying Hypothesis for Alzheimer’s Disease

**DOI:** 10.3389/fgene.2020.00390

**Published:** 2020-05-07

**Authors:** Gwendolyn E. Kaeser, Jerold Chun

**Affiliations:** Sanford Burnham Prebys Medical Discovery Institute, La Jolla, CA, United States

**Keywords:** Alzheimer’s disease, mosaicism, somatic gene recombination, amyloid cascade hypothesis, gencDNA, amyloid precursor protein, APP

## Abstract

The recent identification of somatic gene recombination(SGR) in human neurons affecting the well-known Alzheimer’s disease (AD) pathogenic gene, amyloid precursor protein (APP), has implications for the normal and the diseased human brain. The amyloid hypothesis has been the prevailing theory for sporadic AD (SAD) pathogenesis since the discovery of *APP* gene involvement in familial AD and Down syndrome. Yet, despite enormous scientific and clinical effort, no disease-modifying therapy has emerged. SGR offers a novel mechanism to explain AD pathogenesis and the failures of amyloid-related clinical trials, while maintaining consistency with most aspects of the amyloid hypothesis and additionally supporting possible roles for tau, oxidative stress, inflammation, infection, and prions. SGR retro-inserts novel “genomic complementary DNAs” (gencDNAs) into neuronal genomes and becomes dysregulated in SAD, producing numerous mosaic *APP* variants, including DNA mutations observed in familial AD. Notably, SGR requires gene transcription, DNA strand-breaks, and reverse transcriptase (RT) activity, all of which may be promoted by well-known AD risk factors and provide a framework for the pursuit of new SGR-based therapeutics. In this perspective, we review evidence for *APP* SGR in AD pathogenesis and discuss its possible relevance to other AD-related dementias. Further, SGR’s requirement for RT activity and the relative absence of AD in aged HIV -infected patients exposed to RT inhibitors suggest that these Food and Drug Administration (FDA)-approved drugs may represent a near-term disease-modifying therapy for AD.

## Genomic Mosaicism at the *APP* Locus

We first speculated that SGR might exist in the brain based upon the expression of immunological recombination genes, as described over a quarter century ago for recombination activating gene-1 (Chun et al., 1991) and later, non-homologous end-joining genes (Gao et al., 1998). Subsequent studies to identify somatically generated genomic mosaicism in the human brain identified chromosomal aneuploidies that represent large CNVs (Rehen et al., 2001). The application of newer technologies including fluorescence-activated nuclear sorting (Rehen et al., 2005; Westra et al., 2010) and single-cell sequencing expanded the discovery of somatically arising genomic mosaicism forms, revealing an immense diversity of DNA sequence differences present among single cells (reviewed in Rohrback et al., 2018). This includes Jackson Pollock-like displays reflective of enormous single-cell transcriptome diversity in the brain (Lake et al., 2016, 2018) that is consistent with genomic mosaicism. Neuronal genomic mosaicism takes many forms including aneuploidies, CNVs, single nucleotide variations (SNVs), and long interspersed nuclear element 1 (LINE1). Some of these have been associated with neurodegenerative (including AD) and neuropsychiatric disorders, which have been reviewed extensively and will not be the subject of this perspective (Arendt et al., 2009; Leija-Salazar et al., 2018; Rohrback et al., 2018; Shepherd et al., 2018; Iourov et al., 2019; Potter et al., 2019).

Although the existence of genomic mosaicism is now established, its functions are less clear. Roles in transcriptomic regulation (Kaushal et al., 2003), cell survival (Peterson et al., 2012), and neural circuits (Kingsbury et al., 2005) have been reported, and others have speculated on the importance of genomic mosaicism in the creation of neuronal diversity (Rehen et al., 2001, 2005; Muotri and Gage, 2006; Gericke, 2008), yet these general phenomena did not reveal effects on specific genes or DNA alterations that might be analogous to V(D)J recombination in the immune system (Papavasiliou and Schatz, 2002). However, a candidate gene emerged when we observed increases in a major sub-type of mosaicism called “DNA content variation” (Westra et al., 2010) in SAD neurons of the prefrontal cerebral cortex, where SAD neurons contained ∼500 megabase pairs more DNA than the non-diseased controls (Bushman et al., 2015). We reasoned that the increase could affect *APP*, a key gene in AD pathogenesis that is causal in familial AD and Down syndrome through mutations and, in particular, CNVs: mosaically increased *APP* CNVs in SAD brains may drive pathology. This possibility was confirmed using multiple approaches including PNA-FISH, small-population qPCR, and single-neuron qPCR, which demonstrated that somatic and mosaic changes to the *APP* locus were enriched in SAD neurons over non-diseased controls and were not associated with trisomy of chromosome 21 (Bushman et al., 2015). Interestingly, PNA-FISH targeting individual *APP* exons and exon–exon copy number discordance by single-cell qPCR suggested that the physical arrangement of *APP* CNVs could be non-uniform (Bushman et al., 2015).

**FIGURE 1:**
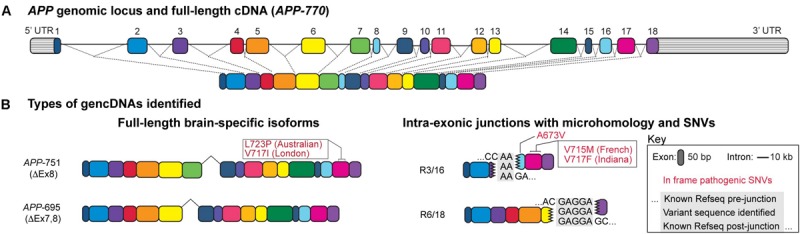
Structure of a gencDNA. **(A)** The *APP* genomic locus and the exons within a full-length cDNA. **(B)** Two types of gencDNAs were identified in both RNA and DNA: full-length brain-specific isoforms (APP-751 and APP-695) and truncated sequences with intra-exonic junctions and microhomology domains (R3/16 and R6/18). Known pathogenic SNVs were also identified in some variants (in-frame examples include Australian, London, French, Indiana, and A673V; shown in red). Figure modified from Lee et al. (2018).

Additional studies confirmed this possibility and revealed SGR at the *APP* locus (Figure 1A), occurring as variant *APP* coding sequences that lacked introns and were akin to complementary DNA (cDNA) sequences except that they were present in genomic DNA and were therefore termed “gencDNAs” (Figure 1B) (Lee et al., 2018). These novel gencDNAs were further characterized by intra-exonic junctions with shared microhomology regions between the two joined exonic regions. Identical forms were also documented in mRNAs. The formation of *APP* gencDNAs *in vitro* required *APP* transcription, DNA strand breakage, and RT activity. Neuronal SGR represents a novel mechanism to produce genomic mosaicism that has functional implications, particularly for AD pathogenesis and therapeutics, while suggesting a more general paradigm underlying sporadic brain diseases through dysregulated SGR of both known and unknown pathogenic genes.

## SGR Affecting *APP* Is Dysregulated in Sad Brains

At least 12 distinct approaches, including non-targeted and unbiased methods, were used to identify and validate somatic mosaic events at the *APP* locus (Bushman et al., 2015; Lee et al., 2018; Lee et al., 2019). SGR was identified in both normal and diseased brains but appears to be dysregulated with disease, resulting in dramatic increases to both the number and the form of *APP* gencDNAs in SAD neurons. Novel approaches were utilized, including DISH and high-fidelity, long-read sequencing to establish disease alterations.

DISH was developed by modifying BaseScope (Advanced Cell Diagnostics, Fremont, CA, United States) technology that can detect SNVs (Baker et al., 2017) and exon:exon junctions in RNA but was adapted for use on genomic DNA. Probes were designed to target multiple gencDNA sequences, including the exon16:exon17 junction and the intra-exonic junction formed by the microhomology fusion of exon 3 to exon 16 (Figure 1B). Several parallel approaches were employed, including sense and antisense probes that demonstrated DNA specificity (*vs.* RNA that is not recognized by sense-strand probes), targeted restriction enzyme digestion that effectively destroyed the DNA target locus and dependent signal, DISH double-labeling that indicated that gencDNA loci are distinct from the endogenous alleles, and the use of synthetic targets in cell culture that confirmed probe specificity (Lee et al., 2018). SAD brains exhibited an average of 1.2–1.8 gencDNAs per nucleus, with 60–70% of prefrontal SAD cortical neuronal nuclei having at least one signal. In contrast, the control brains averaged 0.4 gencDNAs per nucleus, and only 25% of nuclei had at least one signal. The three- to fourfold increase in SAD gencDNA number was consistent throughout all biological and technical replicates (six SAD and six non-diseased brains; three experiments per brain). Notably, detection by this technique is limited to the targeted exon:exon sequence or intra-exonic junction and, therefore, does not capture the full diversity of possible gencDNAs, including full-length or more complex structural variants.

The second novel approach to assess gencDNAs and identify disease-related differences was high-fidelity, long-read sequencing with Pacific Biosciences’ single-molecule real-time circular consensus sequencing (SMRT CCS or “PacBio sequencing”) (Eid et al., 2009; Hebert et al., 2018), which is capable of identifying SNVs with 99.999999% confidence. *APP*-targeted PCR products were amplified from neurons (five SAD and five non-diseased brains) and sequenced. These experiments revealed enormous gencDNA diversity involving thousands of unique species. Importantly, gencDNA sequences changed significantly with disease, despite identical PCR targeting that involved amplification with exon 1 and 18 primers (myriad other species may exist). The SAD brains had 10 times more unique reads per neuronal nucleus, and we identified 45 unique intra-exonic junctions in SAD brains, contrasting with just 20 unique intra-exonic junctions found in non-diseased brains despite using ∼70% more neuronal nuclei. Most remarkably, PacBio sequencing identified 11 mosaic SNVs in or around the Aβ encoding region that are considered to be disease-causing in familial AD, present only in SAD neurons. The results from DISH and PacBio sequencing together confirm that normal human neurons display a baseline level of *APP* gencDNAs that is increased and fundamentally altered in number and form with AD, including the formation of pathogenic SNVs. Independent support for gencDNAs was recently published by an unrelated laboratory (Lee et al., 2019; Park et al., 2019). Preliminary analyses of these data identified diverse integration sites for gencDNAs on multiple chromosomes (Lee et al., 2019) and are consistent with DISH signals that were distinct from wild-type (chromosome 21) alleles (Lee et al., 2018).

## Molecular Diversity Produced by SGR May Link Multiple Ad Hypotheses

**FIGURE 2:**
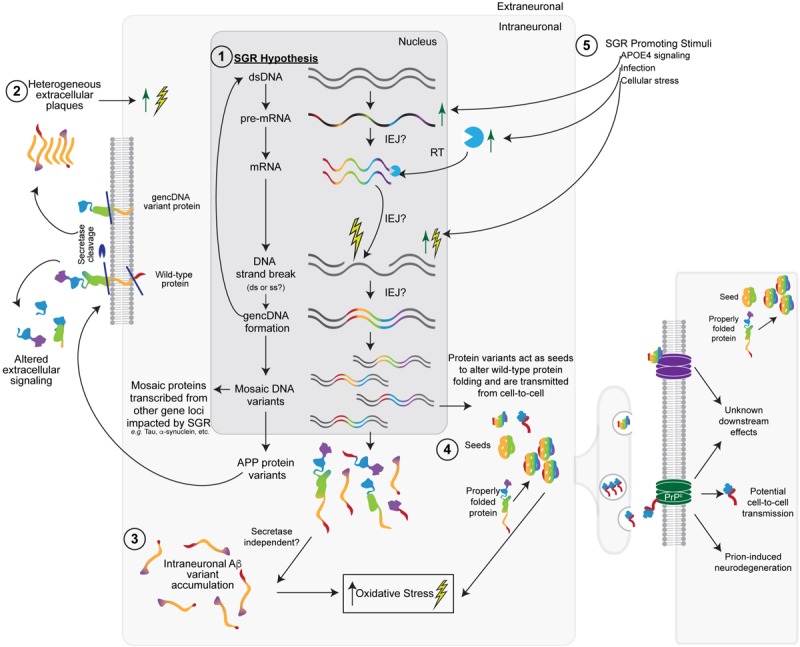
The somatic gene recombination (SGR) hypothesis for SAD. **(1)** Dysregulated SGR of *APP* through the insertion of reverse-transcribed mRNA leads to mosaic genomic *APP* variants that result in variant proteins with a number of downstream effects. Other gene loci may also be impacted by SGR. **(2)** In keeping with the Aβ-hypothesis, some variants would be transported to the cellular membrane, where wild-type, SNV-containing, and gencDNA variant proteins may or may not be cleaved by the traditional secretase pathways to produce heterogeneous extracellular plaques and altered extracellular signaling pathways. **(3)** Intracellular variants may also accumulate without the need for secretase cleavage. The accumulation of intraneuronal Aβ variants likely increases cellular (oxidative) stress, leading to an increase in DNA strand breaks, the insertion of gencDNAs, and the production of variants, creating a feed-forward loop that promotes a disease. **(4)** Variant proteins may also act as “seeds,” which alter the conformation of wild-type APP or other gencDNA variant proteins to create more aggregates. These may be propagated from cell-to-cell and cause prion-like transfer and neurodegeneration through the possible involvement of prion protein receptors (PrP). **(5)** Various stimuli (e.g., APOE4, infection, and cellular stress) could promote SGR *via* actions at multiple steps, including increased *APP* transcription.

Somatic gene recombination likely has normal functions; however, it appears to be dysregulated in AD, most likely during the proposed cellular phase of AD (critically reviewed in De Strooper and Karran, 2016). SGR could create variant *APP* sequences that become translated into heterogeneous populations of APP variant and Aβ-like proteins—in addition to serving as more classical secretase substrates to generate Aβ—that could result in myriad downstream biochemical processes, as was reported for AD. SGR of *APP* could have effects on primary, secondary, tertiary, or quaternary protein structure and therefore could have a vast array of functional effects, including those related to prions. The heterogeneity of *APP* forms produced by SGR invokes modification of the amyloid hypothesis to integrate this new feature while still maintaining decades of supportive observations. SGR also accounts for experimental discrepancies and clinical trial failures. In doing so, it may unify other hypotheses of SAD etiology and pathogenesis *via* a modified amyloid hypothesis (Figure 2). Other pathogenic actions of SGR, such as those produced by the integration of mobile elements, may also be relevant. The initial views on the implications of SGR in AD (Castro et al., 2019; Lee and Chun, 2019) are expanded upon next.

### Amyloid Cascade Hypothesis Modified by Somatic Gene Recombination

The amyloid cascade hypothesis (or the amyloid hypothesis) has been the predominant AD theory for decades (Hardy and Higgins, 1992; Hardy and Selkoe, 2002; Selkoe and Hardy, 2016), having emerged through the identification of amyloid-β (Aβ) as the plaque-forming peptide from AD and Down syndrome brains ∼35 years ago (Glenner and Wong, 1984a, b), which allowed the subsequent identification of *APP* as the gene locus responsible for Aβ (Goldgaber et al., 1987; Tanzi et al., 1987b). The strongest evidence for the involvement of *APP* and its cleavage product Aβ in AD comes from familial AD and Down syndrome studies. Familial AD exhibits typical Mendelian inheritance of mutations or CNVs in *APP* or mutations in the secretase genes, PSEN1 (Sherrington et al., 1995) and PSEN2 (Levy-Lahad et al., 1995; Rogaev et al., 1995) that alter Aβ processing and lead to early-onset AD. *APP* shows a clear gene dosage effect, where three copies of *APP* in Down syndrome (Wiseman et al., 2015) or rare familial AD cases (Rovelet-Lecrux et al., 2006; Sleegers et al., 2006; Hooli et al., 2012) are sufficient to produce AD neuropathology and/or symptomology. In the amyloid hypothesis, the accumulation of Aβ in the brain and its aggregation into plaques result in downstream processes that lead to hyperphosphorylation of tau, resultant neurofibrillary tangles (NFTs), synaptic dysfunction, cell death, and ultimately AD.

One major criticism of the amyloid hypothesis is the timing of plaque deposition. Aβ plaques do not necessarily correlate well with cognitive impairment, and many individuals have abundant Aβ deposits at death and yet were cognitively normal antemortem (Crystal et al., 1988; Katzman et al., 1988; Troncoso et al., 1996). These plaques tend to be more diffuse with lower levels of Aβ oligomers (Esparza et al., 2013), which suggests that pathogenic plaques sequester toxic oligomers (Selkoe and Hardy, 2016). Additionally, there is evidence that the duration of plaque deposition is more predictive of SAD (Insel et al., 2017) rather than the presence of plaques *per se*. Another major criticism of the amyloid hypothesis has been its failure to yield any disease-modifying therapies despite the many clinical trials targeting Aβ components (Carlsson, 2008; Cummings et al., 2014; Anderson et al., 2017; Mehta et al., 2017; Burki, 2018; Egan et al., 2018). These discrepancies and others have led to calls to abandon or fundamentally rethink the amyloid hypothesis (Herrup, 2015; Morris et al., 2018).

However, these major discrepancies may be explained by SGR. SGR may generate diverse APP protein products, including Aβ and Aβ-like molecules, on a continuum of toxicity within plaques and as soluble proteins. Therefore, SGR of *APP* incorporates supportive data for the amyloid hypothesis in SAD by vastly expanding the gene forms, including those containing pathogenic SNVs, and resulting protein products associated with amyloid (Figure 2). These products would access downstream pathogenic cellular mechanisms observed in familial AD but doing so mosaically and somatically in SAD. SGR affecting *APP* also provides an opportunity to reconsider clinical trial failures. All therapeutic antibodies against Aβ are monoclonal, which target distinct epitopes of a conserved amino acid sequence of Aβ (Arndt et al., 2018), and many antibodies are effective at clearing the plaques formed by this amino acid sequence. However, SGR creates myriad different *APP* variant genes, transcripts, and predicted amyloid proteins that may not be recognized by mono-specific Aβ-antibodies. These variant species would therefore remain in the brain in various potential forms (e.g., other plaques, fibrils, prions, and soluble products). Notably, some forms arising from gencDNAs will share conserved epitopes that may be accessed by mono-molecular agents to affect a subset of gencDNAs providing partial efficacy. Similarly, SGR may create products that do not require secretase cleavage, a view supported by the small size of some variant genes and RNA transcripts (Figure 2). Taken together, SGR maintains the central genetic importance of *APP* in familial forms of AD and Down syndrome but significantly extends it to account for SAD without requiring germline changes in *APP.*

Any AD hypothesis must account for statistical relationships to AD risk genes (Kunkle et al., 2019). In this regard, SGR could be augmented by risk genes like APOE4, the major risk allele for AD (Saunders et al., 1993; Strittmatter et al., 1993) that has been shown to increase *APP* transcription and Aβ deposition (Huang et al., 2017; Liu et al., 2017). This function of APOE4 is highly consistent with the SGR hypothesis, where *APP* transcription was shown to be required for gencDNA formation in culture and in J20 mouse neurons. Increases in *APP* transcription could therefore increase the probability of *APP* SGR occurring, and gencDNA production could lead to SGR-dependent “seeds” that promote toxic plaque deposition. Other risk genes could similarly interface with SGR to produce somatic, disease-promoting genomic changes. Notably, PSEN1 variants were not identified using the same detection pipeline that identified *APP* gencDNAs. One possibility is that the gain-of-function end-points that appear to occur for APP are not relevant to the promotion of AD by PSEN1 mutations, a view consistent with the scientific literature that identifies loss-of-function rather than gain-of-function mechanisms (Kelleher and Shen, 2017; Sun et al., 2017).

### Tau Hypothesis Compatibility With SGR

Tau is a microtubule-associated protein that becomes hyperphosphorylated in disease and can aggregate to form NFTs, the second major pathological hallmark of AD (Braak and Braak, 1991; Braak et al., 2006). Tau pathology is closely correlated with neurodegeneration and clinical symptoms (Ossenkoppele et al., 2016; Scholl et al., 2016; Okamura and Yanai, 2017) and may be a key initiator of stressors leading ultimately to cell death in both the Aβ and tau hypotheses (Selkoe and Hardy, 2016; Kametani and Hasegawa, 2018).

Somatic gene recombination is consistent with the tau hypothesis in two distinct and non-mutually exclusive ways. First, SGR generates APP protein variants that could alter tau phosphorylation and processing: a function proposed for Aβ (Rapoport et al., 2002; Dolan and Johnson, 2010; Jin et al., 2011; Moore et al., 2015). Second, SGR might act on the gene for tau—*MAPT*—in the same manner as *APP*, thereby creating myriad and mosaic *MAPT* variants (Figure 2). *MAPT* mutations are known to cause autosomal dominant forms of frontotemporal lobar degeneration (FTLD) and Parkinson’s disease (PD), with over 40 pathogenic mutations identified to date (Rademakers et al., 2004; Ghetti et al., 2015), and SGR could generate related SNVs. There is some debate on whether germline *MAPT* mutations also represent an increased risk for developing AD; however, a recent meta-analysis of a subset of SNVs reported a significantly increased risk for AD that was furthered by APOE4 carrier status (Zhou and Wang, 2017). If *MAPT* were mosaically altered in SAD brains, but perhaps in different cells or brain regions, it might contribute to AD progression, and importantly, explain the high co-morbidity between AD and PD/other proteinopathies (Kovacs et al., 2013; Brenowitz et al., 2017; Kapasi et al., 2017); further study is warranted.

### Prion Hypothesis Relevance to SGR

Prions are misfolded proteins that are able to transmit disease in a fashion similar to an infection, *via* transfer of proteins from cell to cell (Watts and Prusiner, 2018). Evidence has been steadily mounting for the involvement of prions in SAD and other neurodegenerative diseases (e.g., PD and FTLD) in which misfolded proteins are prone to accumulation. Both Aβ (Jaunmuktane et al., 2015; Olsson et al., 2018) and tau (Holmes and Diamond, 2014; Alonso et al., 2016; Kaufman et al., 2016) have been implicated as prion proteins in AD. The enormous potential protein heterogeneity encoded by SGR gene variants is well suited to create DNA sequences encoding mutant prion-like proteins (Figure 2). Such proteins may have seeding effects, leading to the misfolding of normal APP and Aβ—or other SGR-derived proteins—which could then act as prion-like transmissible agents. Were this to occur, SGR variants may impact neighboring cells, perhaps *via* the prion protein receptor itself (Lauren et al., 2009; Gimbel et al., 2010), thereby amplifying the spread of pathogenic SGR products. This mechanism might enable propagation throughout the AD brain to promote the documented neuroanatomical progression of plaques and tangles (Arnold et al., 2013). SGR might also enable the identification of key pathological amino acid sequences in prion-like proteins.

### Inflammation and Cellular Stress Hypotheses and SGR

There are multiple hypotheses for SAD that incorporate some component of the inflammation pathway, oxidative stress, biometal accumulation, and/or mitochondrial dysfunction. These mechanisms likely combine to accelerate neurodegeneration. However, there is debate about whether these processes cause neurodegeneration or are the result of it (Andersen, 2004). Indeed during an inflammatory response, glia produce high levels of free radicals that promote cellular damage and augment neuroinflammation (Solleiro-Villavicencio and Rivas-Arancibia, 2018); thus, the mechanisms underlying such hypotheses are likely to involve a multifaceted, cyclical mechanism of neurodegeneration.

As a class, transposable elements have complex roles in cellular stress (Horvath et al., 2017) that may be emulated or impacted by gencDNAs. Cellular stress causes nucleic acid oxidation which often results in strand breaks. DNA strand breaks were shown to be required for SGR retro-insertion and gencDNA formation in cell culture (Lee et al., 2018). It is therefore possible that a feed-forward mechanism exists where cellular stress causes the strand breaks that enable SGR of *APP* gencDNAs, whose products would, in turn, increase oxidative stress (Figure 2). Additionally, both DNA and RNA oxidation occur in AD brains (Nunomura et al., 1999, 2012); the resultant DNA strand breaks may promote dysregulated gencDNA retro-insertion and could contribute to the formation of intra-exonic junctions.

### Trisomy 21 Hypothesis and SGR

Trisomy of chromosome 21 has long been associated with AD through Down syndrome and the first identification of APP, leading to early hypotheses that SAD might be caused by trisomy 21. This hypothesis was rigorously tested in 1987 and no duplication of chromosome 21 (St George-Hyslop et al., 1987) or the *APP* gene (Tanzi et al., 1987a) was found in bulk samples of SAD brains. Reports on linkage between global mosaic trisomy 21 and SAD have been reviewed elsewhere (Potter, 1991; Potter et al., 2016, 2019). It is notable that mosaic aneuploidies involving all chromosomes are found throughout the normal vertebrate brain, including humans, independent of AD (reviewed in Arendt et al., 2009; Leija-Salazar et al., 2018; Rohrback et al., 2018; Shepherd et al., 2018; Iourov et al., 2019; Potter et al., 2019). While some studies have reported an increase in brain aneuploidies associated with AD (Iourov et al., 2009; Arendt et al., 2010; Yurov et al., 2014), others have not identified disease associations (Thomas and Fenech, 2008; Westra et al., 2009; Bushman et al., 2015). Critically, sampling issues affect all studies of aneuploidy because of the minute fraction of interrogated cells utilized compared to the total number of cells within the brain. Interestingly, increased gene transcription could theoretically increase the probability of SGR for a given gene, in support of a link between chromosomal gains that promote transcription and SGR.

### Infection Hypothesis and SGR

The infection or pathogen hypothesis proposes that viral, fungal, and/or bacterial infections may trigger AD. These hypotheses are based on reports of the presence of viruses, fungi, or bacteria (or their remnant signatures) within the SAD brain (Hill et al., 2014; Itzhaki et al., 2016; Harding et al., 2017). Some viruses, including human immunodeficiency virus (HIV) and hepatitis B virus (HBV) (Mastroeni et al., 2018), as well as certain bacteria (Lampson et al., 2005), possess demonstrated RT activity—a requirement for SGR. In addition, the vast diversity of *APP* gencDNAs could conceivably produce proteins that could bind to and possibly neutralize infectious agents by analogy to immunoglobulins in the immune system (Eimer et al., 2018). However, the causality, specificity, and presence of these infectious agents require further study, as underscored by reports of bacterial contamination as artifacts in human microbiome studies (reviewed in Eisenhofer et al., 2019).

### The SGR Hypothesis in AD

The preceding discussion outlines concepts and hypotheses that could be accessed by SGR. Normally, *APP* SGR acts first upon mRNAs transcribed from the wild-type locus producing varied *APP* gencDNAs *via* an RNA intermediate and RT activity, requiring DNA strand breaks to enable retro-insertion. These gencDNA sequences then retro-insert into genomic DNA, generating cDNA-like sequences that lack introns. They may be full-length DNA copies of known *APP* splice variants (*APP* 571 and 695) or appear as truncated forms containing intra-exonic junctions. The insertion sites appear most commonly outside of wild-type loci, based upon DISH and initial insertion site analyses, with relatively few cells containing one or more copies. Normal SGR may represent a form of cellular memory, where activity-dependent transcription and DNA breaks of multiple etiologies enable the incorporation of gencDNAs, particularly as preferred and already-spliced forms that could later be re-expressed using similar or different promoters.

In disease, dysregulation of SGR occurs. It appears to involve coordinate actions of at least three SGR components: gene transcription, RT activity, and DNA strand breaks. Dysregulation then produces myriad numbers and forms of gencDNAs that could be neurotoxic *via* retro-insertion (as documented for other mobile elements; Horvath et al., 2017), other non-coding disruptions of RNA and DNA, and/or pathogenic APP proteins with altered primary, secondary, tertiary, or quaternary structure that would impact the functionality of APP, Aβ, and prion-like proteins. Known risk genes could be involved, for example, with APOE4 increasing *APP* transcription (Huang et al., 2017) that would, in turn, increase SGR. In this view, some classes of risk factor genes would promote SGR actions on AD “driver” or causal genes. Similarly, increased inflammation or oxidative stress would increase DNA strand breaks, resulting in more gencDNA retro-insertions into new, potentially deleterious genomic locations. Additional risk factors would increase pathogenic gencDNA variants in a feed-forward loop to increase gencDNA production that includes pathogenic SNVs known from familial cases, passing through a disease threshold. Other pathogenic SNVs not compatible with life (and familial AD manifestation)—and other genes—could also be produced in SAD, while familial AD and Down syndrome pathology may also involve SGR, which could explain the decades of life still required to produce AD in these genetic disorders. SGR could generate prion-like sequences producing toxic protein accumulations in neuroanatomically defined patterns. Critically, SGR utilizes RT that appears to create SNVs through imprecise template copying and also identifies an accessible therapeutic strategy using Food and Drug Administration (FDA)-approved RT inhibitors (Lee and Chun, 2019).

## SGR and Other Brain Diseases

The existence of SGR in the normal and the AD brain could potentially unify mechanisms for neurological and possibly neuropsychiatric sporadic brain diseases, where somatic, mosaic changes in DNA sequences generate pathogenic loci. Most other neurodegenerative and neuropsychiatric disorders, including AD-related dementias like FTLD and PD dementia/Lewy body dementia, present most commonly as a sporadic disease. SGR in theory could act on any number of gene loci, including those identified in familial disease to produce mosaic genomic variations that drive sporadic disease. Notably, new mutations in known pathogenic genes as well as in unrecognized genes could be somatically and mosaically altered in the brain, which again might be incompatible with life if present in the germline. SGR dysregulation could also explain the multiple decades it takes for most neurodegenerative diseases to progress as well as patient-to-patient variability in disease progression, wherein the generation and the accumulation of pathogenic gene variants occur mosaically over time. Similarly, SGR can explain the comorbidity of mixed dementias, where ∼50–75% of patients with dementia have neuropathology from at least two of the AD-related dementias (Kovacs et al., 2013; James et al., 2016; Brenowitz et al., 2017; Kapasi et al., 2017). In this scenario, SGR could act on different genes within the same brain, affecting various cell types and neuroanatomical regions.

## SGR and Reverse Transcriptase Activity

The origin of brain RT activity is not yet clear. RTs were first discovered in retroviruses (Baltimore, 1970; Temin and Mizutani, 1970). However, a more likely source of endogenous RT activity in humans resides in the genome, within what was once called “junk DNA” (Ohno, 1972) and includes LINE1 sequences, human endogenous retrovirus (HERVs), and sequences encoding telomerases. LINE1 sequences account for ∼17% of the human genome and include over 500,000 copies (Lander et al., 2001). Two open reading frames within LINE1 are ORF1, thought to encode a high-affinity RNA binding protein, and ORF2 that encodes an RT and an endonuclease. A vast majority of these sequences are thought to be inactive; however, some have been shown to enable retro-transposition in cancer and have been implicated as a driver of evolution (Lee et al., 2012). LINE1 has also been hypothesized to contribute to neuronal diversity by disrupting existing genes or DNA elements upon re-insertion of LINE1 sequences during neurogenesis (Muotri et al., 2005), a concept that remains under active investigation (Evrony et al., 2012 *vs*. Upton et al., 2015). Another 8% of the genome is made up of HERVs that contain a possible RT within its *pol* gene, albeit with limited expected activity in the human genome (Nelson et al., 2003, 2004; Thomas et al., 2018). Human telomerase (encoded by the TERT gene) also has RT activity (Leao et al., 2018) and may further provide an RT source, as could other unknown enzymes.

### The Clinical Potential of SGR Inhibition in AD and Other Brain Diseases

The demonstrated involvement of RT activity in SGR implicates its inhibition as a possible AD preventative and/or therapeutic intervention. Critically, multiple FDA-approved RT inhibitors have been developed for the treatment of HIV (and later, HBV), with over three decades of continuous use as part of combination anti-retroviral therapies, which may provide real-world evidence of their efficacy in the prevention and/or treatment of AD. The number of treated HIV-infected patients who are also at risk of SAD (being ∼65 years or older) currently number up to ∼80,000–100,000 patients in the United States (CDC, 2018), which would yield an expected AD prevalence in the thousands (10% of all persons age 65 or older have AD; Alzheimer’s Association, 2019). A surveillance of these patients has occurred for over a decade in anticipation of an increase of AD in HIV-infected patients (Alisky, 2007), yet only one documented AD/HIV-infected case has appeared in the peer-reviewed literature (Turner et al., 2016). The limitations of *post hoc* epidemiology are acknowledged, and prospective AD clinical trials are needed. However, even if confounders resulted in these numbers being off by a factor of 100, there would still be a significant difference between the number of reported HIV-infected SAD cases *vs*. the expected prevalence of SAD in this age group. Since approved RT inhibitors have over 30 years of real-world human safety data, legal, off-label prescription by a licensed physician represents a promising option for AD patients where no effective and safe therapy currently exists.

## Conclusion

Genomic mosaicism in the human brain is a biological fact that manifests through multiple forms of DNA sequence changes within single cells, from aneuploidies through SNVs. The recent discovery of SGR acting on *APP* provides functionality for genomic mosaicism through actions on a single gene, with both normal and disease implications. Normally, SGR may act as a form of cellular memory (Crick, 1984; Davis and Squire, 1984), where transcriptional activity and resulting DNA breaks may enable the retro-insertion of gencDNAs ready for re-expression as pre-spliced and varied mRNAs and diverse protein products: a form of long-lasting memory. SGR may also resemble forms of genomic “streamlining” that have been documented through phylogeny and contribute to species evolution (Roy and Gilbert, 2006). The dysregulation of SGR produces disease through increased numbers and forms of toxic gencDNAs, as illustrated by somatic, genomic changes documented in SAD brains. Importantly, the SGR hypothesis in AD does not reject the amyloid hypothesis outright but rather incorporates major features to modify the hypothesis while also accommodating other distinct hypotheses and explaining discrepancies in the scientific and clinical trial literature through the generation of *APP* variants and downstream molecular diversity. SGR presents a new source of potential therapeutics, some with near-term implications for the treatment and/or prevention of AD by use of FDA-approved medicines targeting endogenous brain RTs, an approach supported by human epidemiological data on older HIV-infected patients. SGR and its roles in AD represent a new step toward understanding the functions of genomic mosaicism in the normal, aging, and diseased brain.

## Author Contributions

GK and JC wrote this manuscript.

## Conflict of Interest

JC is the co-founder of Mosaic Pharmaceuticals.

GK declare that the research was conducted in the absence of any commercial or financial relationships that could be construed as a potential conflict of interest.

## Abbreviations

AD: Alzheimer’s disease
APP: amyloid precursor protein
CNV: copy number variation
DISH: DNA *in situ* hybridization
gencDNA: genomic complementary DNA
PNA-FISH: peptide nucleic acid fluorescent *in situ* hybridization
RT: reverse transcriptase
SAD: sporadic AD
SGR: somatic gene recombination.
